# Controls of a superconducting quantum parametron under a strong pump field

**DOI:** 10.1038/s41598-021-90874-4

**Published:** 2021-06-01

**Authors:** Shumpei Masuda, Toyofumi Ishikawa, Yuichiro Matsuzaki, Shiro Kawabata

**Affiliations:** Research Center for Emerging Computing Technologies (RCECT), National Institute of Advanced Industrial Science and Technology (AIST), 1-1-1, Umezono, Tsukuba, Ibaraki 305-8568 Japan

**Keywords:** Qubits, Theoretical physics

## Abstract

Pumped at approximately twice the natural frequency, a Josephson parametric oscillator called parametron or Kerr parametric oscillator shows self-oscillation. Quantum annealing and universal quantum computation using self-oscillating parametrons as qubits were proposed. However, controls of parametrons under the pump field are degraded by unwanted rapidly oscillating terms in the Hamiltonian, which we call non-resonant rapidly oscillating terms (NROTs) coming from the violation of the rotating wave approximation. Therefore, the pump field can be an intrinsic origin of the imperfection of controls of parametrons. Here, we theoretically study the influence of the NROTs on the accuracy of controls of a parametron: a cat-state creation and a single-qubit gate. It is shown that there is a trade-off relationship between the suppression of the nonadiabatic transitions and the validity of the rotating wave approximation in a conventional approach. We also show that the tailored time dependence of the detuning of the pump field can suppress both of the nonadiabatic transitions and the disturbance of the state of the parametron due to the NROTs.

## Introduction

Parametric phase-locked oscillators^1^, which are also called parametrons^2^, can store binary digital information as the phase of the self-oscillation when they are driven via a periodic modulation of their circuit element. Parametrons were actually operated as classical bits in digital computers in 1950s and 1960s until the transistor acquired the solid stability. More recently, parametrons were revived in the nanoelectromechanical, optical and the superconducting circuit systems. Basic bit operations have been demonstrated in a nanoelectromechanical system using a electromechanical resonator^3^, and the Ising machine based on optical parametron has been proposed^4^. To see the quantum nature of the parametron, the nonlinearity should be sufficiently large compared to the decay rate. The nonlinearity smaller than the decay rate gives rise to the appearance of classical dynamics of the system^5^. The quantum regime with the nonlinearity larger than the decay rate has been studied theoretically^6–8^ and experimentally^9,10^. We consider this quantum regime in this paper.

The parametron was applied to the qubit readout^11,12^ in circuit QED architectures which are promising platform of quantum information processing^13–15^. Quantum annealing^16–18^ and universal quantum computation^19^, which utilize the quantum nature of parametrons in a superconducting circuit, have been proposed. Recently, the bias-preserving gates^20^ and single-qubit operations^21^ were studied theoretically and experimentally. Exponential increase of the bit-flip time with the cat size was also observed^22^.

Under the pump field oscillating at approximately twice its natural frequency, a superconducting quantum parametron (we refer parametron hereafter) can work as a qubit in contrast to transmons and flux qubits which do not require an oscillating pump field to realize an effective two-level system.

The decay from the parametron causes the decoherence of the qubit states^23^. In order to avoid the decoherence, we need controls much faster than the decay rate. For such rapid controls, we require a large pump field to avoid unwanted nonadiabatic transitions^19^. However, the strong pump field can be an origin of the degradation of qubit operations. Such a trade-off relationship has been overlooked in earlier studies on the parametron.

In this paper, we study the effect of the strong pump field to the operations of a parametron in the quantum regime assuming that the operation time is much shorter than the coherence time. First, in order to quantitatively assess the feasibility of superconducting parametron for quantum applications, we study the effect of the unwanted non-resonant rapidly oscillating terms (NROTs) in the Hamiltonian on the accuracy of the creation of a cat state. It is shown that there is a trade-off relationship between the suppression of the nonadiabatic transitions and the validity of the rotating wave approximation in a conventional approach^16,19^. Second, we also show that the tailored time dependence of the detuning of the pump field can suppress both the nonadiabatic transitions and the disturbance of the state of a parametron due to the NROTs. Finally, we study the effect of the NROTs on an $R_x$ gate.

## Model

**Figure 1:**
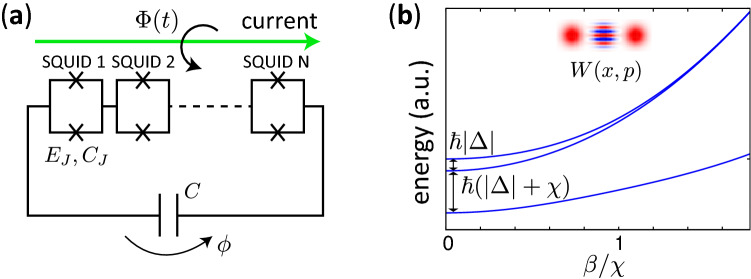
(**a**) Schematic of a superconducting quantum parametron. $E_J$ and $C_J$ are the Josephson energy of a single SQUID and the capacitance of a single Josephson junction, respectively. *C* denotes the capacitor shunting the SQUID array. $\phi$ is the overall phase across the junction array. $\Phi (t)$ is the external magnetic flux threading the SQUIDs. (**b**) Energy level diagram as a function of pump strength for $\Delta <0$ and $\chi >0$. Because $\Delta <0$, the highest energy level is the vacuum state for $\beta =0$. The inset is a typical image of the Wigner function of the highest energy level for large $\sqrt{2\beta /\chi }(\simeq 2.5)$. The top two curves overlap as $\beta$ is sufficiently large.

We consider a parametron composed of a SQUID-array resonator with *N* SQUIDs (Fig. 1a) which was implemented in Ref.^10^. The effective Hamiltonian of the system is represented as^10^

1 $$\begin{aligned} H= 4E_C n^2 - NE_J[\Phi (t)] \cos \frac{\phi }{N}, \end{aligned}$$

where $\phi$ and *n* are the overall phase across the junction array and its conjugate variable, respectively. $E_J$ is the Josephson energy of a single SQUID. The effective Hamiltonian with a single degree of freedom, $\phi$, is valid when the Josephson energy $E_J$ is much greater than the charging energy of a single junction^24^. $E_C$ is the resonator’s charging energy including the contributions of the junction capacitance $C_J$ and the shunt capacitance *C*, and can be extracted from measurements and also can be calculated with finite-element capacitance simulations^10^. The Josephson energy is periodically modulated by the external magnetic flux, $\Phi (t)$, threading the SQUIDs as $E_J(t)=E_J+\delta E_J \cos \omega _p t$.

Taking into account up to the 4th order of $\phi /N$ in Eq. (1), we obtain an approximate Hamiltonian

2 $$\begin{aligned} \frac{H}{\hbar }= & {} \omega _c^{(0)} \Big ( a^\dagger a + \frac{1}{2} \Big ) - \frac{\chi }{12} (a + a^\dagger )^4 + \Big [ - \frac{N\delta E_J}{\hbar } + 2\beta (a + a^\dagger )^2 - \frac{2\chi \beta }{3\omega _c^{(0)}} (a + a^\dagger )^4 \Big ] \cos \omega _p t, \end{aligned}$$

where $\omega _c^{(0)} = \frac{1}{\hbar }\sqrt{8E_CE_J/N}$, $\chi =E_C/\hbar N^2$ and $\beta = \omega _c^{(0)} \delta E_J/ 8E_J$. Here, $\beta$ corresponds to the pump strength. The annihilation operator *a* is related to *n* and $\phi$ as $n = -in_0(a-a^\dagger )$ and $\phi = \phi _0 (a+a^\dagger )$ with $n_0^2=\sqrt{E_J/32 N E_C}$ and $\phi _0^2 = \sqrt{2NE_C/E_J}$. For the expansion of Eq. (1), we considered the parameter regime, where $\phi _0/N = 2\sqrt{\chi /\omega _c^{(0)}}$ is sufficiently smaller than unity so that the approximation is valid. We took into account up to the forth order of $\phi /N$ to see the effect of the Kerr nonlinearity, which is important for a parametron. We neglect the last term in Eq. (2) assuming $\chi \beta \ll \omega _c^{(0)}$, and drop c-valued terms to obtain

3 $$\begin{aligned} \frac{H}{\hbar } = \omega _c^{(0)} a^\dagger a - \frac{\chi }{12} (a + a^\dagger )^4 + 2\beta (a + a^\dagger )^2 \cos \omega _p t. \end{aligned}$$

Moving into a rotating frame at the frequency of $\omega _p/2$, the Hamiltonian is written as

4 $$\begin{aligned} \frac{H}{\hbar }= & {} \Big (\omega _c^{(0)}-\omega _p/2 \Big ) a^\dagger a - \frac{\chi }{12} (a e^{-i\frac{\omega _p}{2}t} + a^\dagger e^{i\frac{\omega _p}{2}t} )^4 + 2\beta (a e^{-i\frac{\omega _p}{2}t} + a^\dagger e^{i\frac{\omega _p}{2}t})^2 \cos \omega _p t. \end{aligned}$$

When we neglect all the oscillating terms such as $a^2e^{-2i\omega _p t}$ which are called NROTs, we obtain an approximate Hamiltonian (rotating wave approximation),

5 $$\begin{aligned} \frac{H_{\mathrm{RWA}}}{\hbar } = \Delta a^{\dagger} a - \frac{\chi }{2} a^{\dagger} a^{\dagger} a a +\beta (a^2 + a^{\dagger 2}), \end{aligned}$$

where $\Delta = \omega _c^{(0)}-\chi -\omega _p/2$. We compare the results for the Hamiltonians in Eqs. (4) and (5) in the following sections. We neglect the decay and the dephasing to highlight the effect of the NROTs assuming that the decay and the dephasing time is sufficiently longer than the duration of the controls.

Figure 1(b) shows a schematic of the energy level diagram of the Hamiltonian (5). The vacuum state is the highest energy level in the rotating frame when $\beta =0$. The highest and the second highest energy levels for sufficiently large $\beta /\chi$ are represented as

6 $$\begin{aligned} |\varphi _0\rangle\,\simeq\, & {} \frac{|-\alpha \rangle + |\alpha \rangle }{\sqrt{2}},\nonumber \\ |\varphi _{1}\rangle\,\simeq\, & {} \frac{|-\alpha \rangle - |\alpha \rangle }{\sqrt{2}}, \end{aligned}$$

respectively, with coherent states, $|-\alpha \rangle$ and $|\alpha \rangle$, where $\alpha = \sqrt{(2\beta +\Delta )/\chi }$^25^, and $|\Delta |$ is much smaller than $\beta$. These coherent states , $|-\alpha \rangle$ and $|\alpha \rangle$, can be used as a qubit for quantum annealing and universal quantum computation^16,19^. Thus, the creation of predetermined states such as cat states in Eq. (6) is of importance for quantum information processing.

In this paper, we consider the case that $\Delta \le 0$. If $\Delta$ is positive, the vacuum state is not the highest energy level in the rotating frame when $\beta =0$, and the vacuum state is driven to a state different from $|\varphi _0\rangle$ as the pump field is ramped^8^.

## Results

We examine the effect of the NROTs on the creation of a cat state, $|\varphi _0\rangle$, and on an accuracy of a single-qubit gate along the *x* axis ($R_x$ gate). We solve the time-dependent Schrödinger equation with a fourth-order Runge-Kutta integrator with the time step of 0.025 fs in the following numerical simulations.

### Creation of a cat state

We assume that the system is in the vacuum state and $\beta =0$ at $t=0$; and $\beta$ is gradually increased for $0\le t\le T$. The quantum adiabatic theorem states that the system remains in the highest energy level if $\beta$ is increased slowly enough. Thus, the population of the highest energy level, $p_0$, is unity if the evolution is completely adiabatic. We set the time dependence of $\beta$ as

7 $$\begin{aligned} \beta (t) = \left\{ \begin{array}{cc} \beta _0 t / T \ &{} \mathrm{for} \ \ 0\le t \le T,\\ \beta _0 \ &{} \mathrm{for} \ \ t > T. \end{array} \right. \end{aligned}$$

(We consider a linear ramp of $\beta$ for simplicity.) We define the fidelity of the control as $p_0(t)$ for $t>T$.

**Figure 2:**
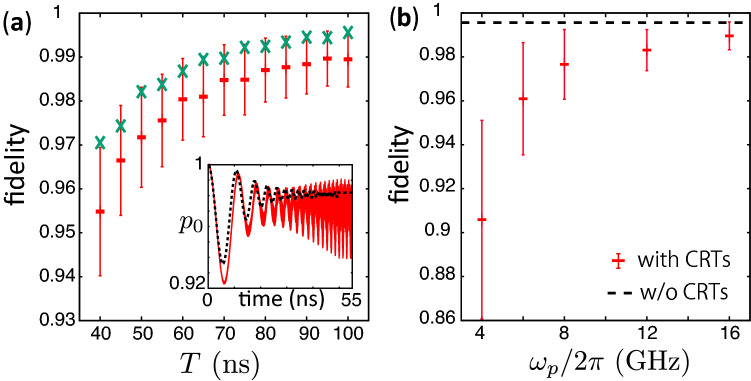
(**a**) Fidelity of the creation of a cat state as a function of *T* for the dynamics with (red) and without (green) the NROTs, where the error bars represent the standard deviation calculated using the data for $t>T$. The used parameters are $\beta _0/2\pi = 200$ MHz, $\Delta /2\pi =-6.7$ MHz, $\omega _p/2\pi =16$ GHz and $\chi /2\pi =68$ MHz. The inset shows the time evolution of $p_0$ for $T=50$ ns. The red solid and the black dashed curves are for with and without the NROTs, respectively. We chose $\Delta$ to be the same as the previous study^10^. (**b**) Fidelity as a function of $\omega _p/2\pi$ for $T=100$ ns. We use the same value for $\beta _0$, $\chi$ and $\Delta$ as (**a**), while $\omega _c^{(0)}$ is changed so that $\Delta$ is unchanged (Note that $\omega _c^{(0)} = \omega _p/2 + \Delta + \chi$). The dashed line corresponds to the dynamics without the NROTs.

Figure 2a shows the fidelity of the control as a function of *T*. The fidelity for short *T* is lowered due to unwanted nonadiabatic transitions in the dynamics without NROTs. In the dynamics with the NROTs, the fidelity is even lower and keeps fluctuating after the ramp of the pump field. The standard deviation of the fluctuation of $p_0$ for $t>T$ is considerably large even for $T=$ 100 ns where the nonadiabatic transitions are negligible. The fluctuation becomes large when *T* is short because of the large population of the lower levels. Figure 2b shows the fidelity as a function of $\omega _p$. In this numerical simulation, $\omega _c^{(0)}$ is changed with $\omega _p$ so that the detuning is fixed. It is seen that, as $\omega _p$ increases, the fidelity is increased and the fluctuation of $p_0$ is suppressed. This comes from the fact that the rotating wave approximation becomes more accurate as we increase $\omega _p$ and $\omega _c^{(0)}$.

**Figure 3:**
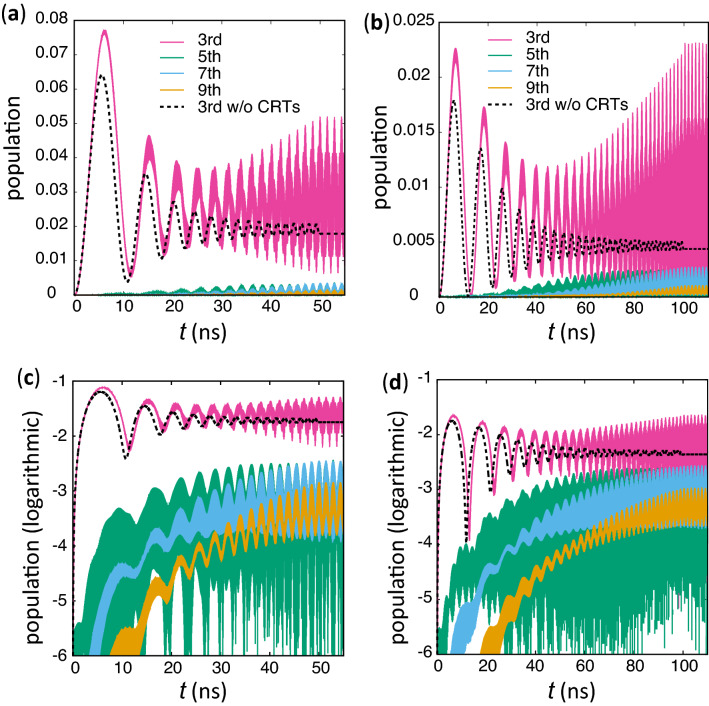
Time dependence of the population of the third, fifth, seventh and the nineth highest levels during the cat-state creation for $T=50$ ns (**a**) and 100 ns (**b**). The population of the second, fourth, sixth, $\cdots$ levels is vanishing because of the difference of the parity. The dotted curves represent the population of the third highest level in the dynamics without NROTs. The used parameters are the same as Fig. 2a. (**c**) and (**d**) are the same things as (**a**) and (**b**), respectively, but with the vertical axis in the logarithmic scale.

The time dependences of the population of lower levels are shown for $T=50$ ns and 100 ns in Fig. 3a,c and Fig. 3b, d respectively. In the case without the NROTs, the third highest level is populated due to the nonadiabatic transition while the population of the other lower levels are approximately zero (*e*.*g*., the population of the fifth highest level is less than $10^{-5}$ and $10^{-6}$ at $t=T$ for the parameters used in Fig. 3a,c and Fig. 3b, d respectively). The population of the second, fourth, sixth, $\cdots$ levels is vanishing because of the parity difference from the highest level. On the other hand, the other lower levels with the same parity as the highest level are also populated in the dynamics with the NROTs as apparently seen in Fig. 3. The fluctuating population of the third highest level is higher than that without the NROTs for the both values of *T*. The oscillation of the populations saturates for $t>T$, when $\beta$ is constant.

We discuss the significance of our results here. It is worth mentioning that we need a condition of $\langle -\alpha |\alpha \rangle \simeq 0$ to use the parametron as a qubit, and so $\beta /\chi$ should be sufficiently large. (The overlap, $\langle -\alpha |\alpha \rangle$, becomes negligible when $\beta /\chi$ is sufficiently large because $\langle -\alpha | \alpha \rangle = \exp [-2|\alpha |^2]$^16^ and $\alpha =\sqrt{(2\beta +\Delta )/\chi }$.) For this purpose, we could decrease $\chi$, but this leads us to a smaller energy gap between the eigenenergies of the Hamiltonian, which could induce more nonadiabatic transitions. An alternative approach to satisfy $\langle -\alpha |\alpha \rangle \simeq 0$ while supressing the nonadiabatic transitions could be a increase of $\beta$. However, as we showed in this subsection, a large $\beta$ could be another source of error due to the violation of the rotating wave approximation. Therefore, in the conventional approach, there is a trade-off relationship between the suppression of the nonadiabatic transitions and the validity of the rotating wave approximation, which was often overlooked in earlier works.

#### Suppression of nonadiabatic transitions

In order to overcome the trade-off relationship discussed in the previous subsection, we examine a way to enhance the fidelity of the creation of a cat state based on the time-dependent detuning^26^. We show that the fluctuation of the population of the target state due to the NROTs and the nonadiabitc transitions are greatly suppressed without increasing $\beta$ nor decreasing $\chi$.

In this method, we set the initial detuning large and decrease it to zero as

8 $$\begin{aligned} \Delta (t) = \left\{ \begin{array}{clc} \Delta _0 (1- t / T) \ &{} \mathrm{for} \ \ 0\le t \le T,\\ 0 \ &{} \mathrm{for} \ \ t > T. \end{array} \right. \end{aligned}$$

The pump is ramped following Eq. (7). We set the initial detuning $\Delta _0/2\pi =-67$ MHz. The time-dependent detuning can be implemented by controlling $\omega _c^{(0)}$ depending on $E_J$ which can be controlled with the magnetic flux. Unwanted resultant change in $\beta$ can be compensated by changing $\delta E_J$. Alternatively, the time-dependent frequency of the pump field can be used for the implementation of the time-dependent detuning.

**Figure 4:**
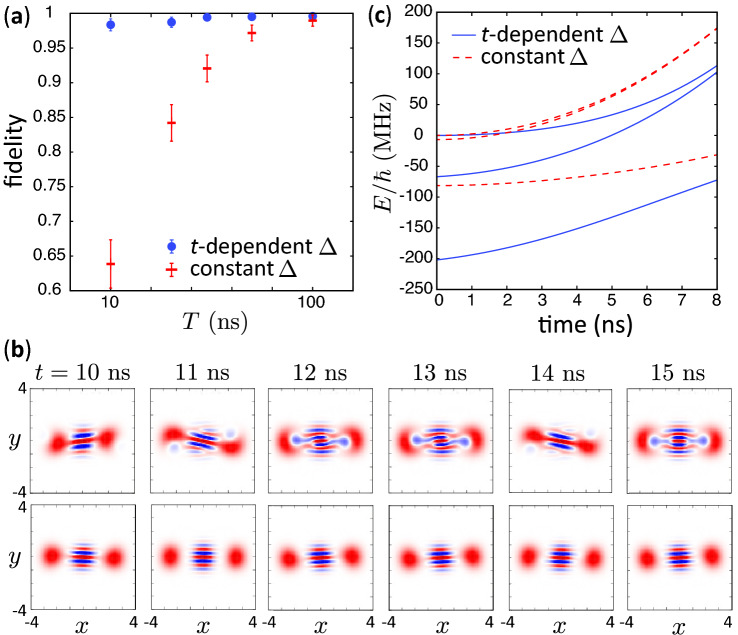
(**a**) Fidelity of the creation of a cat state as a function of *T* with the time-dependent detuning in Eq. (8) (blue circles) and the constant detuning of $\Delta /2\pi =-6.7$ MHz (red bars). The NROTs are taken into account in the both dynamics. The error bars represent the standard deviation which is calculated using the data for $t>T$. The used parameters are $\Delta _0/2\pi =-67$ MHz, $\beta _0/2\pi = 200$ MHz, $\omega _p/2\pi =16$ GHz and $\chi /2\pi =68$ MHz. (**b**) Wigner functions for $t\ge T (=10\,\mathrm{ns})$ in the controls with the constant (upper figures) and the time-dependent (lower figures) detunings. The other parameters are the same as (**a**). (**c**) The three highest eigenenergies of instantaneous $H_{\mathrm{RWA}}$ in Eq. (5) for the constant and the time-dependent detuning for $T=20$ ns. The other parameters are the same as (**a**).

Figure 4a represents the fidelity of the creation of a cat state as a function of *T*. The modified method gives the fidelity considerably higher than the one with the constant detuning. We have obtained the fidelity of more than 0.995 with the modified method for $T=50$ ns while the average fidelity for the control with the constant detuning is approximately 0.97. We emphasize that the fluctuation of the fidelity is suppressed in the modified method as seen in the error bars of Fig. 4a. We attribute this to the fact that the population of the lower levels are much smaller than the case with the constant detuning. Note that the NROTs, which couples the highest level to the other levels, weakly influence to the population of the highest level, when the population of the other levels are small. Figure 4b represents the Wigner function^16^ for $t\ge T (=10\,\mathrm{ns})$ in the controls with the constant and the time-dependent detunings. The Wigner function is disturbed and time dependent in the control with the constant detuning for $t\ge T$, while in the modified method it is approximately stationary and coincides with that of the highest energy level of $H_{\mathrm{RWA}}$. The results for the controls with different values of $\Delta _0$ are shown in Supplementary Section S1.

Figure 4c shows the three highest eigenenergies of instantaneous $H_{\mathrm{RWA}}$ in Eq. (5) for the constant and the time-dependent detuning. The reader may consider that the nonadiabatic transitions occur when *t* is large because the interval between the highest and the second highest levels become small. However, such transition does not occur because of the parity difference. The major population transfer is from the highest level to the third highest level.

The enhancement of the fidelity in the modified method is explained as follows. It is known that the adiabatic condition:

9 $$\begin{aligned} h_{mn}(t) = \hbar |\langle \varphi _n(t) | {{\dot{\varphi }}}_m(t) \rangle | / |E_n(t)-E_m(t)| \ll 1 \end{aligned}$$

should be satisfied to suppress the nonadiabatic transition between levels *m* and *n*, where $E_m$ is an eigenvalue of the instantaneous $H_{\mathrm{RWA}}$, and $m\ne n$. The state of the highest level of $H_{\mathrm{RWA}}$ changes drastically from the zero photon state to a superposition of Fock states as the pump is ramped in the small pump regime. The introduced large detuning in the small pump regime makes slow the rate of the change of the highest level, and makes the denominator of Eq. (9) large. Thus, the dynamics is well approximated by the adiabatic dynamics (nonadiabatic transitions are suppressed). On the other hand, the rate of the change of the highest level is slow for the large pump regime compared to the small pump regime. Therefore, the detuning can be gradually turned off.

**Figure 5:**
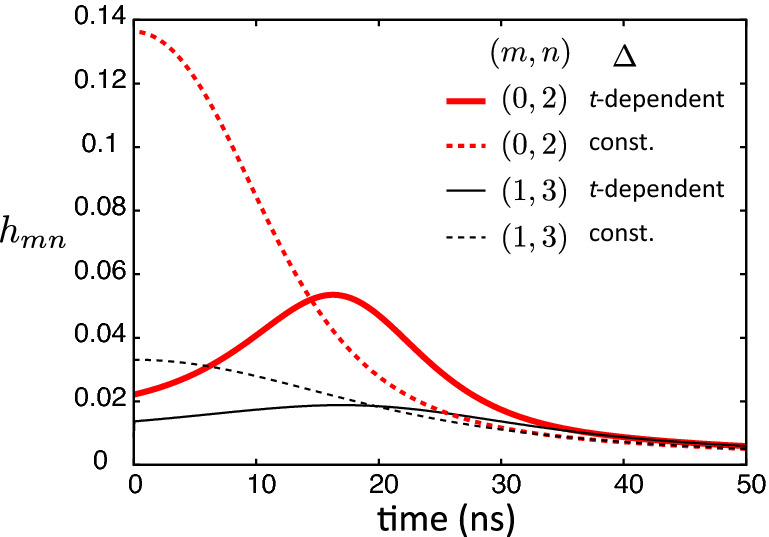
Time dependence of $h_{mn}$ for the creation of a cat state for $T=50$ ns with the time-dependent detuning in Eq. (8) and the constant detuning. Other parameters are the same as Fig. 2a.

Figure 5 shows the time dependence of $h_{mn}$ during the creation of a cat state with the time-dependent detuning in Eq. (8) and the constant detuning. It is seen that $h_{mn}$ for the time-dependent detuning are smaller than the one for the constant detuning around $t=0$, and the peaks of $h_{mn}$ for the time-dependent detuning is lower than the maximum value for the control with the constant detuning.

Now, a comment is in order. Using larger constant detuning also can improve the fidelity of the creation of a cat state. However, finite $\Delta$ causes $R_x$ gate of the parametron as explained in the following section because $\Delta$ increases the gap between the highest level and the second highest level of the parametron. Although using larger pump strength can decrease the gap, it increases the disturbance of the state due to NROTs. Therefore, it is favorable to make $\Delta$ zero at the end of the creation of a cat state from the point of view of the information processing.

The decay from the parametron, which decoheres the qubit state, is an another origin of the imperfection of the control. The effect of the decay to the creation of a cat state is examined in Supplementary Section S2, although we focus mainly on the effect of NROTs in this paper.

Before moving to the next section, we surmmarize the trade-off relations and explain the role of our method. Creation of a cat state should be followed by some other controls such as gate operations and a measurement in applications. Therefore, the speed of creation of cat state should be sufficiently faster than the decay rate for practical purposes. Moreover, for quantum computation, such a fast control is essential to improve the clock frequency. $\beta /\chi$ should be increased rapidly, and its final value should be sufficiently large to use $| -\alpha \rangle$ and $| \alpha \rangle$ as qubit state. Then, we have the trade-off relations: 1. Choosing smaller $\chi$ causes more nonadiabatic transitions due to a smaller energy gap between the eigenenergies; 2. Making $\beta$ larger causes more decrease and larger fluctuation of the fidelity of the control due to the effect of NROTs. The modified method with the time dependent detuning can increase the fidelity by decreasing the nonadiabatic transitions and can suppress the fluctuation of the fidelity.

### $R_x(\frac{\pi }{2})$ gate

A pulsed detuning realizes a rotation of a parametron around the *x* axis^19^. The detuning enlarges the energy difference between the highest and the second highest levels of the instantaneous Hamiltonian. Thus, the states obtain the different dynamical phases, which give rise to a $R_x$ gate. This scheme of the $R_x$ gate differs from the one which utilizes the time-dependent pump strength in Ref. ^21^.

We examine the degradation of the fidelity of the $R_x(\frac{\pi }{2})$ gate due to the NROTs using the pulsed detuning given by

10 $$\begin{aligned} \Delta (t) = \left\{ \begin{array}{cl} \Delta _0 \sin ^2 ({\pi t}/{T_g}) \ &{} \ \mathrm{for} \ \ 0\le t \le T_g,\\ 0 &{} \ \mathrm{for} \ \ t >T_g, \end{array} \right. \end{aligned}$$

where $T_g$ is the gate time and $\Delta _0$ is optimized for $R_x(\frac{\pi }{2})$ gate (the angle of rotation is determined by $\Delta _0$). The other parameters are fixed during the control. The initial state is set to be

11 $$\begin{aligned} |\Psi (0)\rangle = (|\varphi _0\rangle + |\varphi _1\rangle )/\sqrt{2}\simeq |-\alpha \rangle . \end{aligned}$$

The fidelity of the gate is defined by the population of the target state,

12 $$\begin{aligned} |\Psi _{\mathrm{tar}}\rangle = (|\varphi _0\rangle - |\varphi _1\rangle )/\sqrt{2}\simeq |\alpha \rangle \end{aligned}$$

at $$t=T_g$$.

**Figure 6:**
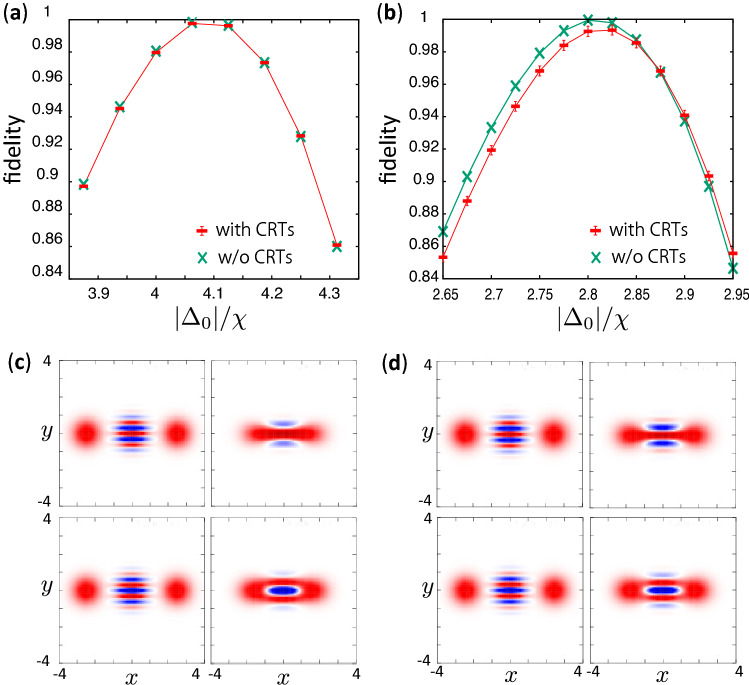
Fidelity of $R_x(\frac{\pi }{2})$ gate. The used parameters are $\beta /2\pi =53$ MHz, $\chi /2\pi =17$ MHz for (**a**) and $\beta /2\pi =200$ MHz, $\chi /2\pi =68$ MHz for (**b**). We used $\omega _p/2\pi =16$ GHz and $T_g=100$ ns for the both panels. The error bars represent the standard deviation calculated using the data for $t>T_g$. (**c**) and (**d**): Wigner function of the highest (upper panels) and the second highest levels (lower panels) of $H_{\mathrm{RWA}}$ in Eq. (5). The left and the right panels correspond to $\Delta =0$ and $\Delta =\Delta _0$, where $|\Delta _0|/\chi =4.1$ for (**c**) and 2.8 for (**d**), respectively. The other parameters used in (**c**) and (**d**) are the same as (**a**) and (**b**), respectively.

We consider two sets of $(\beta ,\chi )$ which give approximately the same $\alpha$. Figure 6a,b show the fidelity of the $R_x(\frac{\pi }{2})$ gate for the both parameter sets with and without the NROTs. In the case without NROTs, the both parameter sets give the fidelity of approximately unity. The maximum fidelity for the smaller $\beta$ and $\chi$ is approximately the same as the case without the NROTs [Fig. 6a] (The difference between them is less than 0.1%). On the other hand, the fidelity for the parameter set with larger $\beta$ and $\chi$ is degraded when the NROTs are taken into account as seen in Fig. 6b. This means that, smaller parameter set is more suitable to decrease the disturbance by the NROTs in the $R_x$ gate, although the smaller parameter set tends to induce more nonadiabatic transitions during the creation of the cat state. Fortunately, we have found that the method with Eq. (8) suppresses the nonadiabatic transitions and the fluctuation of the state when we create a cat state, as shown in Fig. 4. Therefore, we can safely choose the smaller parameter set of $\beta$ and $\chi$ to achieve the higher fidelity of $R_x$ gate while the nonadiabatic transitions and the fluctuation of the state during the cat-state creation are still significantly suppressed by using the modified method.

A comment on the intermediate state during the gate operation is in order. The larger parameter set gives small values of $|\Delta _0|/\chi$ and $|\Delta _0|/\beta$ to perform the $R_x(\frac{\pi }{2})$ gate. The required value of $|\Delta _0|/\chi$ is approximately 4.1 and 2.8 for the smaller and the larger parameter sets, respectively. Thus, the intermediate states during the gate operations are different. Figure 6c,d show the Wigner function of the highest and the second highest levels of $H_{\mathrm{RWA}}$ in Eq. (5) for $\Delta =0$ and $\Delta =\Delta _0$. The Wigner function, which is separated in three parts for $\Delta =0$, is connected near the origin for $\Delta =\Delta _0$. It represents that the highest and the second highest levels become closer to the zero photon and the one photon Fock states, respectively. The Wigner function for the larger parameter set is shrunk in the $y-$direction around the origin compared to that for the smaller parameter set because of the difference in $|\Delta _0|/\chi$.

$R_z$ and $R_x$ gates can consist of a universal single-qubit gate set. $R_z$ gates for a parametron can be realized by a drive with a microwave pulse^19^. Because the intensity of the microwave pulse is sufficiently smaller than the pump field, the interplay between the microwave pulse and the NROTs is negligible (see S3 for detail).

## Conclusion

We have quantitatively investigated the effect of the non-resonant rapidly oscillating terms (NROTs) on controls of a parametron. It has been shown that the NROTs cause unwanted population transfer from the qubit levels to the other energy levels, and degrade the fidelity of the cat-state creation. The population transfer is mainly from the highest level to the third highest level when the frequency of the pump field is sufficiently high. However, we can increase the control fidelity by suitably choosing parameters such as the nonlinearity parameter, the pump strength and frequency. Furthermore, starting from large detuning and decreasing it to zero as the pump is ramped, we can greatly enhance the fidelity of the cat-state creation, which we call a modified method. Interestingly, the fluctuation of the population of the target state is suppressed in the modified method. The mechanism of the enhancement of the fidelity has been explained from the viewpoint of the adiabatic condition. Also, we have studied the effect of the NROTs on a $R_x$ gate. The fidelity of the $R_x$ gate depends on the pump strength because of the NROTs. We have shown that smaller pump field and nonlinearity parameter realize higher gate fidelity.

Turning on and off the pump field can be used not only for the cat-state creation but also for transforming a parametron to a transmon for the qubit readout^21^. Therefore, the inverse process of the modified adiabatic method of the creation of a cat state is expected to be useful also for that purpose.

## Supplementary Information

Supplementary Information.
