# Supplementary material for: Controls of a superconducting quantum parametron under a strong pump field
Source: Sci Rep. 2021 Jun 1;11:11459. doi: 10.1038/s41598-021-90874-4 (PMC8169783; doi:10.1038/s41598-021-90874-4)
Supplement: Supplementary file 1 — Supplementary Information. [file 41598_2021_90874_MOESM1_ESM.pdf]

Supplemental information: Controls of a superconducting quantum parametron under a strong pump field

Shumpei Masuda[1,*], Toyofumi Ishikawa[1], Yuichiro Matsuzaki[1] and Shiro Kawabata[1]

[1] Research Center for Emerging Computing Technologies (RCECT), National Institute of Advanced Industrial Science and Technology (AIST), 1-1-1, Umezono, Tsukuba, Ibaraki 305-8568, Japan

* shumpei.masuda@aist.go.jp

S1 Controls with various values of Δ_0

We consider the creation of a cat state with the time-dependent detuning in Eq. (8) for various values of Δ_0. Figure S1 shows the fidelity as a function of Δ_0 for $T = 20$ ns. The fidelity higher than 0.98 is realized for $|\Delta_0|/2\pi > 40$ MHz. (The fidelity for the constant detuning shown in Fig. 4(a) is less than 0.85.) The fidelity does not increase monotonically with respect to Δ_0 for $|\Delta_0|/2\pi > 40$ MHz. We attribute this fluctuation to nonadiabatic transitions due to the rapid change of the detuning.

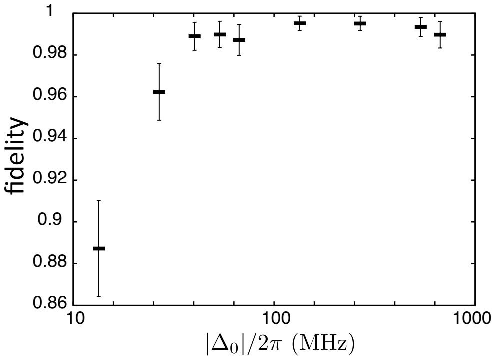

Figure S1. Dependence of the fidelity on $|\Delta_0|$ for the creation of a cat state for $T = 20$ ns with the time-dependent detuning in Eq. (8) . Other parameters are the same as Fig. 4(a).

Figure S2(a) shows the time dependence of h_{02} in Eq. (9) for the control with the time-dependent detuning in Eq. (8) and the control with the constant detuning. h_{02} for the control with the constant detuning is high around $t = 0$, and its maximum value is higher than the peaks of the controls with the time-dependent detuning. The peak for the time-dependent detuning with $\Delta_0/2\pi = -670$ MHz is slightly higher than the one for the control with $\Delta_0/2\pi = -134$ MHz. The time dependence of the population of the third highest level, p_2, is shown in Fig. S2(b). It is seen that p_2 increases around $t = 0$ in the control with the constant detuning. On the other hand, the increase of p_2 occurs later in the controls with the time dependent detuning. The average value of p_2 around $t = 20$ ns is much smaller than the one for the control with the constant detuning. We attribute this time dependence of p_2 to the time dependence of h_{02} which should be small to avoid the nonadiabatic population transfer from the highest level to the third highest level.

S2 Effect of decay

In the main text, we consider controls of which duration is sufficiently shorter than the coherence time. Thus, the decoherence of the system is neglected. When the above condition is not satisfied, the control is degraded by the decoherence. It is known that the decay of the the nonlinear resonator causes the effective dephasing of a parametron. The rate of the phase decay is represented as[1] $\gamma = 2\kappa|\alpha|^2$, where κ is the decay rate of the nonlinear resonator. In this section, we examine the effect of the decay to the two kinds of the creation of a cat state studied in the main text by solving the master equation

$$\dot{\rho} = -i[H_{\rm RWA}, \rho] + \frac{\kappa}{2}\Big([a\rho, a^\dagger] + [a, \rho a^\dagger]\Big), \tag{S1}$$

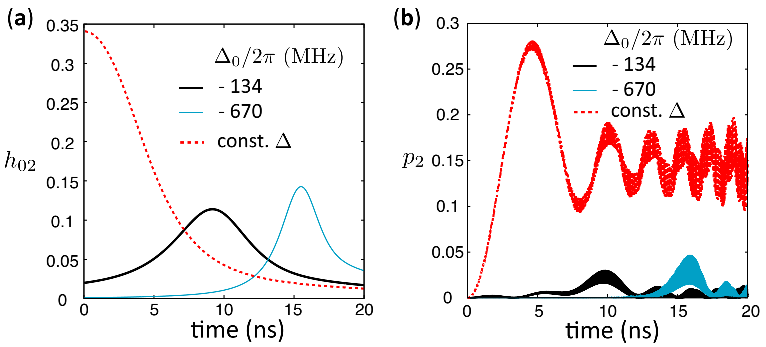

Figure S2. (a) Time dependence of h_{02} for the creation of a cat state for $T = 20$ ns with the time-dependent detuning in Eq. (8) and the constant detuning of $\Delta/2\pi = -6.7$ MHz. We used $\Delta_0/2\pi = -134$ MHz and -670 MHz for the controls with the time-dependent detuning. (b) Time dependence of the population of the third highest level, p_2, in the same dynamics as panel (a). Other parameters are the same as Fig. 4(a).

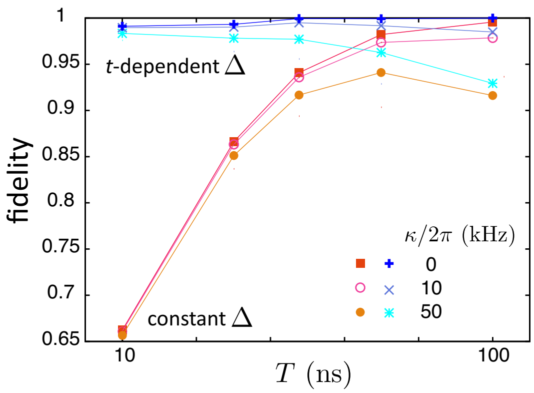

Figure S3. Fidelity of the creation of a cat state as a function of T with the constant detuning and the time dependent detuning. The decay rate, κ, are taken into account in the both dynamics. The solid lines are guide to the eye. Other parameters are the same as Fig. 4(a).

where ρ is the density matrix of the system. The NROTs are not taken into account in this calculation.

Figure S3 shows the fidelity as a function of T for various values of κ. It is observed that the decrease of the fidelity due to the decoherence becomes small as T decreases. For example, the change of the fidelity is less than 0.2 % for the controls with $T = 10$ ns and $\kappa/2\pi = 10$ kHz. In Ref. 2, the amplitude damping time T_1 of 15.5 μs was reported for a superconducting nonlinear resonator, which corresponds to $\kappa/2\pi \simeq 10$ kHz.

Now we take into account both the decay rate, κ, and the NROTs. Figure S4 shows the fidelity of the creation of a cat state for the both controls for $T = 10, 20, 30, 50, 100$ ns. The fidelity is lower compared to the cases where either of the decay or the NROTs are taken into account. Decrease and fluctuation of the fidelity is seen even in the control with the time-dependent detuning for $\kappa/2\pi = 10$ kHz and $T = 10$ ns. We attribute this to the nonadiabatic transitions because the decay becomes more significant if Fock states with higher photon number are populated. Such decease and fluctuation are relatively suppressed for $T = 30$, 50 and 100 for $\kappa/2\pi = 10$ kHz in the control with time dependent detuning.

S3 R_z gate

R_z gate for a parametron using a pulsed microwave was proposed in Ref. 3. Drive of a parametron by a microwave with the frequency of $\omega_p/2$ and the amplitude of $E(t)$ adds a term:

$$H_z(t) = \hbar E(t)(a + a^\dagger) \tag{S2}$$

into Hamiltonian (4) in the rotating frame used in the main text. When $|E(t)|$ is sufficiently small, the parametron is approximately kept in the subspace expanded by $|-\alpha\rangle$ and $|\alpha\rangle$, where we assume that β is constant, $\Delta = 0$, and β/χ is sufficiently

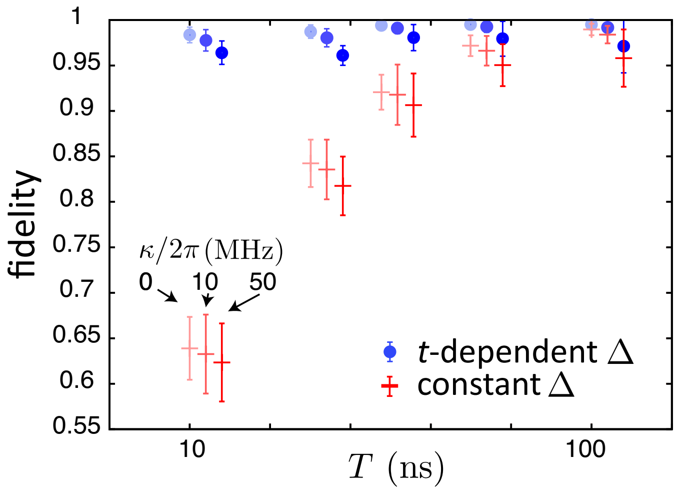

Figure S4. Fidelity of the creation of a cat state as a function of T with the time-dependent detuning in Eq. (8) (blue circles) and the constant detuning of $\Delta/2\pi = -6.7$ MHz (red bars) for $\kappa/2\pi = 0$, 10 and 50 kHz. (The data points for $\kappa/2\pi = 10$ and 50 kHz are shifted horizontally for clarity.) Both the decay rate, κ, and the NROTs are taken into account in the simulation. Other parameters are the same as Fig. 4(a).

large so that the overlap, $\langle -\alpha|\alpha\rangle$, is negligible. The energy of $|\pm\alpha\rangle$ shifts by $\pm 2\hbar E(t)\sqrt{2\beta/\chi}$. The energy shifts give different dynamical phase to $|-\alpha\rangle$ and $|\alpha\rangle$. Thus, the phase difference between the two coherent states results in $R_z(\phi)$ gate at $t = T$ with

$$\phi = 4\sqrt{2\beta/\chi}\int_0^T E(t)dt. \tag{S3}$$

The interplay between the pulse for R_z gate and the NROTs of the pump field can be neglected because $|E(t)|$ is much smaller than β as shown below.

Now, we simulate $R_z(\pi)$ taking into account NROTs of the pump field using $E(t)$ given by[3]

$$E(t) = \begin{cases} \frac{\pi^2}{8T_g\sqrt{2\beta/\chi}}\sin\frac{\pi t}{T_g} & \text{for } 0 \le t \le T_g, \\ 0 & \text{for } t > T_g, \end{cases} \tag{S4}$$

where T_g is the duration of the pulsed field. The pump field is fixed as $\beta = \beta_0$. The initial state is the highest level, $|\varphi_0\rangle$, in Eq. (6). We use the parameter set: $T_g = 10$ ns, $\Delta/2\pi = 0$ MHz, $\beta_0/2\pi = 200$ MHz, $\omega_p/2\pi = 16$ GHz, $\chi/2\pi = 68$ MHz. The peak value of $|E(t)|$ is 24 times smaller than β_0. The $R_z(\pi)$ gate drives the state to the second highest level, $|\varphi_1\rangle$, in Eq. (6). The fidelity of the control is defined by the population of $|\varphi_1\rangle$. The fidelity averaged for $t > T_g$ and the standard deviation of the fluctuation of the fidelity are 0.994 and 0.003, respectively. Figure S5 shows the time dependence of the population of the highest and the second highest levels for $0 < t < 15$ ns. It is seen that the population is transferred from $|\varphi_0\rangle$ to $|\varphi_1\rangle$.

We simulate the dynamics without the pulsed field, $E(t) = 0$, for comparison. In this dynamics, the parametron should stay in $|\varphi_0\rangle$ if there is no NROT. The fidelity is defined by the population of $|\varphi_0\rangle$. The averaged fidelity and the standard deviation of the fluctuation of the fidelity are approximately the same as those with the pulsed field in Eq. (S4). Therefore, the effect of the interplay between the pulsed filed for $R_z(\pi)$ and the NROTs of the pump field can be neglected with the parameters used.

References

1. Puri, S., Boutin, S. & Blais, A. Engineering the quantum states of light in a Kerr-nonlinear resonator by two-photon driving. *npj Quantum Inf.* **3,** 18 (2017).
2. Grimm, A., Frattini, N. E., Puri, S., Mundhada, S. O., Touzard, S., Mirrahimi, M., Girvin, S. M., Shankar, S. & Devoret, M. H., Stabilization and operation of a Kerr-cat qubit. *Nature* **584,** 205-209 (2020).
3. Goto, H. Universal quantum computation with a nonlinear oscillator network. *Phys. Rev.* A **93,** 050301(R) (2016).

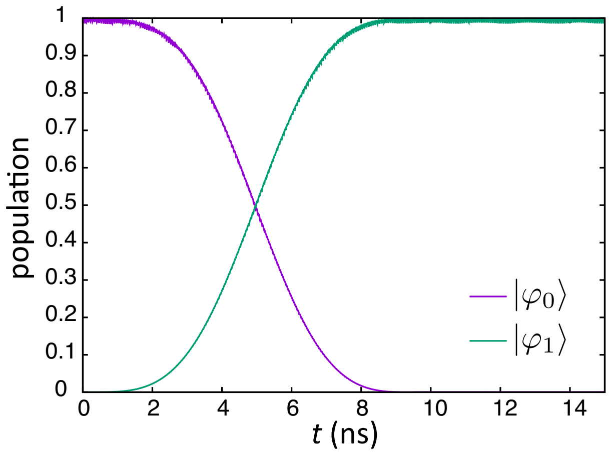

Figure S5. Time dependence of the population of the highest and the second highest levels during the $R_z(\pi)$ gate with $E(t)$ in Eq. (S4). The pump strength is kept constant, $\beta(t) = \beta_0$. The used parameter set is $T_g = 10$ ns, $\Delta/2\pi = 0$ MHz, $\beta_0/2\pi = 200$ MHz, $\omega_p/2\pi = 16$ GHz, $\chi/2\pi = 68$ MHz.
